# Time-of-day variation affects onset but not hematoma size in intracerebral hemorrhage

**DOI:** 10.3389/fneur.2026.1815359

**Published:** 2026-04-13

**Authors:** Franziska Lieschke, Philipp Dorsch, Maximilian Rauch, Mario Balo, Erik Maronde, Christian Grefkes, Ferdinand O. Bohmann

**Affiliations:** 1 Department of Neurology, Goethe University Frankfurt, University Hospital, Frankfurt am Main, Germany; 2 Department of Neurology with Experimental Neurology, Charité Universitätsmedizin Berlin, Corporate Member of the Freie Universität Berlin and Humboldt-Universität Berlin, Berlin, Germany; 3 Faculty of Medicine, Goethe University Frankfurt, Frankfurt am Main, Germany; 4 Goethe University Frankfurt, University Hospital, Institute of Neuroradiology, Frankfurt am Main, Germany; 5 Goethe University Frankfurt, Dr. Senckenberg Institute for Anatomy II, Experimental Neurobiology, Frankfurt am Main, Germany; 6 Medizinische Hochschule Hannover, Institute for Neuroanatomy and Cell Biology, Hannover, Germany

**Keywords:** brain hemorrhage, chronobiology, circadian rhythm, ICH epidemiology, volumetry

## Abstract

**Introduction:**

In acute ischemic stroke, the time of day has been shown to influence the progression of the ischemic core, ultimately impacting patient outcomes. For intracerebral hemorrhage (ICH), data on such an impact on ICH severity are conflicting. Our aim was to identify possible associations between the timing of ICH onset and radiographic and clinical characteristics.

**Methods:**

We conducted a retrospective monocentric study on 381 adult patients with spontaneous ICH who were treated between 2010 and 2024. Patients were categorized by time of symptom onset (morning: 5:00 AM−10:59 AM, midday: 11:00 AM−4:59 PM, evening: 5:00 PM−22:59 PM and night: 23:00 PM−4:59 AM). Primary outcome was ICH volume at the first imaging scan. Secondary outcomes included edema volume, mortality, the presenting syndrome severity and Modified Rankin Scale (mRS) at discharge.

**Results:**

Intracerebral hemorrhage (ICH) onset exhibited a distinct distribution, with peaks around noon and afternoon (~4 PM), and the lowest frequency during nighttime. ICH and edema volumes, mortality, initial clinical severity, and functional outcomes did not differ significantly between onset-time groups. Independent predictors of mortality included age (OR 1.04, 95% CI 1.01–1.07, *p* = 0.03), pre-mRS score (OR 1.37, 95% CI 1.04–1.81, *p* = 0.03), and NIHSS at admission (OR 1.05, 95% CI 1.02–1.09, *p* = 0.002). Unfavorable outcomes (higher mRS at discharge) were associated with pre-mRS (OR 1.33, 95% CI 1.19–1.48, p < 0.001), NIHSS (OR 1.06, 95% CI 1.04–1.07, *p* < 0.001), hypertension (OR 1.49, 95% CI 1.06–2.10, *p* = 0.02), and atrial fibrillation (OR 1.43, 95% CI 1.05–1.94, *p* = 0.02).

**Conclusion:**

In our cohort, ICH onset times peaked during the daytime, however initial ICH and perifocal edema volumes did not differ according to the time of day. Among patients with witnessed and precisely documented ICH onset, early survival and short-term functional outcomes in patients treated at a university hospital with 24-h neurosurgical availability appear to be more strongly influenced by individual patient characteristics such as age and pre-existing conditions than by the timing of symptom onset.

## Introduction

Previous studies have shown that the timing of stroke onset is important for neurological severity, treatment course and functional outcome in acute ischemic stroke (1–3). However, data on such an influence are still insufficiently investigated in intracerebral hemorrhage (ICH). Perilesional edema as a marker of secondary injury typically begins to develop within the first few hours and can expand over several days (4, 5), however multiple studies suggest that the greatest growth occurs in the first 24 h (6–8). This growth is largely driven by both mechanical effects of the hematoma and secondary injury mechanisms such as inflammation, blood–brain barrier disruption, and toxic breakdown products of blood (e.g., hemoglobin and iron), all of which might be influenced by the time of day (9). As most therapeutic approaches target secondary injury mechanisms, initial hematoma and edema sizes are of relevance. The aim of this study was to identify possible associations between the timing of ICH onset and radiographic and clinical characteristics that could impact patient outcomes.

## Methods

The study included consecutive patients aged 18 years or older who were diagnosed with ICH and received treatment at our tertiary care center between 2010 and 2024. To ensure data integrity and consistency, the exclusion criteria comprised incomplete datasets, specifically cases where the symptom onset could not be reliably determined, such as in wake-up strokes or patients with unknown last seen well, as well as cases where a retrospective review revealed a diagnosis other than spontaneous ICH, such as ischemic stroke, vasculitis, trauma, or subarachnoid hemorrhage. Ethical approval for this retrospective study, which involved the anonymized use of routine data without disclosure to third parties, was obtained from the local ethics committee (No. 2024-1901). Hence, informed consent was explicitly not necessary.

We distinguished patients with symptom onset during the morning (5:00 AM−10:59 AM), midday (11:00 AM−4:59 PM), evening (5:00 PM−22:59 PM) and night (23:00 PM−4:59 AM) (10), as calculated from the time of admission and reported delay from symptom onset. We collected information on baseline characteristics, the time of admission, timeframe from symptom onset, previous risk factors, among others. The primary outcome measure was ICH volume (in milliliters).

Secondary outcomes included presence of perifocal edema (in milliliters), intrahospital mortality, the initial severity of stroke symptoms, measured using the National Institutes of Health Stroke Scale (NIHSS), the NIHSS score after 24 h as well as the Modified Rankin Scale (mRS) score at discharge.

Manual volumetric assessments of ICH and perifocal edema were performed in consensus by two neuroradiologists, who were blinded to demographic and clinical information. Measurements were conducted on a Centricity workstation (GE Healthcare), based on the initial neuroimaging studies, using axial non-contrast CT scans (slice thickness 1.25 mm) or axial T2-weighted MRI sequences (slice thickness 5 mm), respectively.

To adjust for confounding, inverse probability weighting (IPW) for patient age, sex and medical history (arterial hypertension, atrial fibrillation, smoking, hypercholesterolemia, any type of previous stroke, alcohol and diabetes mellitus) was applied.

Statistical analysis was performed using GNU R 4.4.1 and GraphPad PRISM 10.3.1. Normally distributed data is depicted as mean ± standard error of the mean (SEM), continuous data without normal distribution as median and interquartile range (IQR), and frequencies of categorical data as percentage. Normality was assessed with the Shapiro–Wilk test. Intergroup differences of the included characteristics were evaluated using the *t*–test, Mann–Whitney *U*-test, χ^2^ or fisher's test, or using the Kruskal–Wallis test if more than two groups were compared. For predictor analysis, ordered multinomial and logistic regression models were performed with inverse probability weights. We considered *p*-values < 0.05 to be statistically significant.

## Results

### Study population

**Figure 1:**
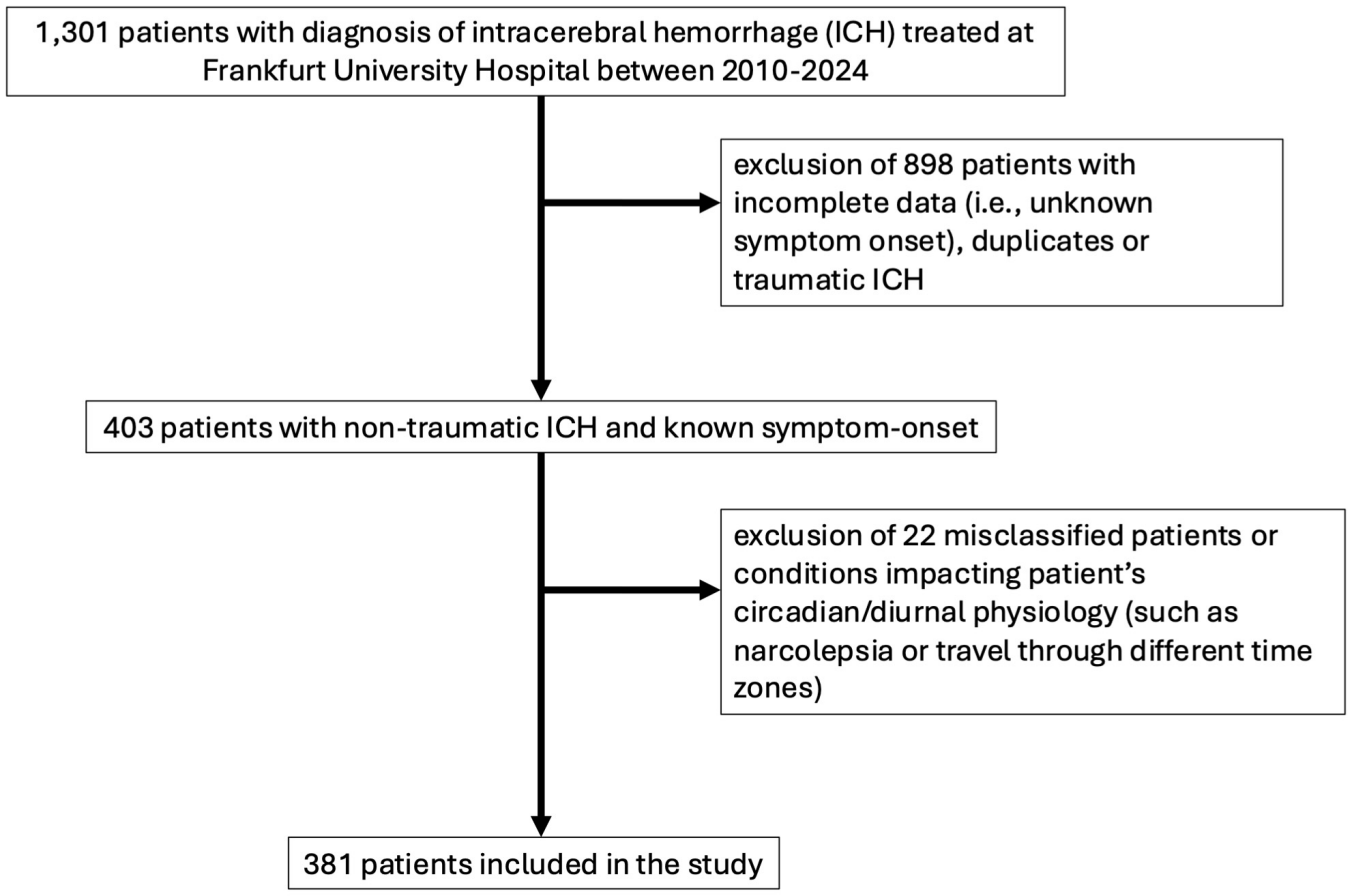
Study flow chart depicting the inclusion and exclusion criteria.

Overall, 1,301 ICH patients were treated at Frankfurt University Hospital between 2010 and 2024. To ensure accurate allocation to the respective time points, only patients with non-traumatic bleeds and a clearly reported symptom onset were included in the following analysis (403 patients). After excluding a further 22 patients who were retrospectively diagnosed with conditions other than ICH, or who had conditions impacting the endogenous circadian physiology (three with vasculitis, five with cerebral venous and dural sinus thrombosis, seven with ischemic stroke, one with subarachnoid hemorrhage, five referrals from the airport where the patients had experienced the event after a long-haul flight involving a time zone shift, and one patient with narcolepsy), the final analysis included 381 patients (see Figure 1).

### Bimodal pattern of ICH incidence

**Figure 2:**
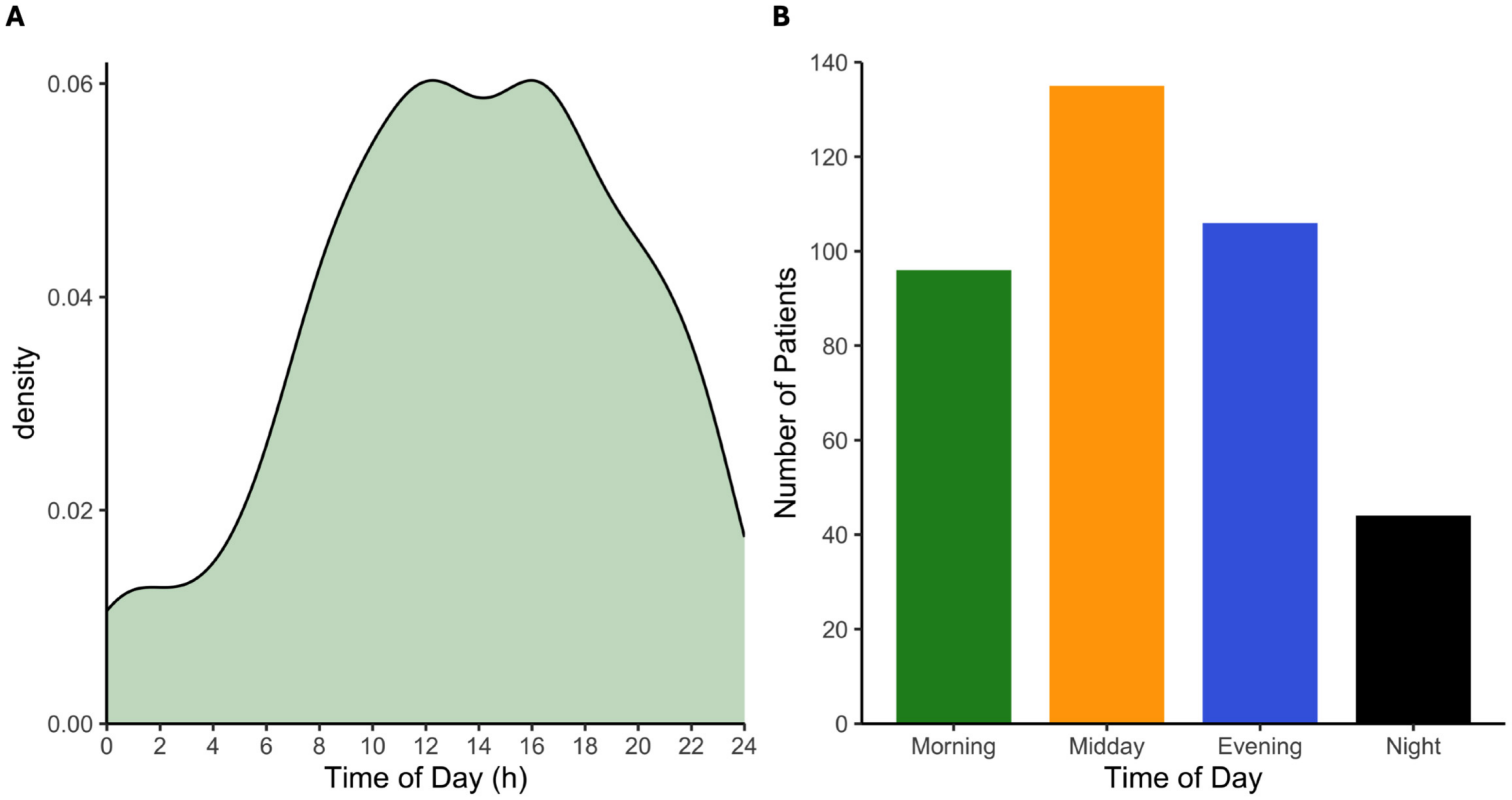
Kernel density estimates of onset times reveals a bimodal distribution, with prominent peaks occurring around noon and a secondary maximum in the afternoon **(A)** with histogram **(B)**. Overall, ICH incidence was higher during daytime hours, with reduced frequencies observed in the evening and nighttime.

In 96 of the patients ICH onset was during the morning (5:00 AM−10:59 AM), in 135 patients, ICH occurred during the midday (11:00 AM−4:59 PM), in 106 during the evening (5:00 PM−10:59 PM), and in 44 during the night (11:00 PM−4:59 AM). The continuous variation in ICH incidence time of day is shown in Figure 2. Onset times displayed a bimodal pattern with peak incidences occurring around noon and in the afternoon (12:15 PM and 4:00 PM, respectively). Only 11.5% of ICH were during the night (11:00 PM−4:59 AM) with minimum incidence occurring at approximately midnight. During night hours, a small peak at 1:45 AM was detectable.

The baseline characteristics in the four time categories are shown in Table 1. Age was higher in morning onset patients than other groups (*p* = 0.03). Times between symptom onset and hospital admission were significantly longer during nighttime (*p* = 0.02). Prevalence of smoking was highest in patients with nighttime ICH onset and lowest in the midday onset group (*p* = 0.04), with a similar pattern seen for hypercholesterolemia (*p* = 0.03).

**Table 1:** Baseline characteristics according to the time of day of ICH onset.

Patient characteristics	All (*n* = 381)	Morning (*n* = 96)	Midday (*n* = 135)	Evening (*n* = 106)	Night (*n* = 44)	*p*-value
Median age (IQR) [years]	71 (58–80)	75 (61–83)	69 (58–80)	71 (57–79)	70 (53–77)	**0.03**
Sex (%)						
Male	230 (60.4)	55 (57.3)	88 (65.2)	58 (54.7)	29 (65.9)	0.3
Female	151 (39.6)	41 (42.7)	47 (34.8)	48 (45.3)	15 (34.1)	
Median pre-mRS (IQR)	1 (0–2)	1 (0–2)	1 (0–2)	1 (0–2)	1 (0–2)	0.85
pre-mRS (%)						
0	158 (42.7)	38 (41.3)	59 (44.7)	44 (42.3)	17 (40.5)	0.73
1	86 (23.2)	22 (23.9)	29 (22.0)	25 (24.0)	10 (23.8)	
2	60 (16.2)	10 (10.9)	24 (18.2)	16 (15.3)	10 (23.8)	
3	41 (11.1)	11 (12.0)	14 (10.6)	13 (12.5)	3 (7.1)	
4	18 (4.9)	7 (7.6)	5 (3.8)	4 (3.9)	2 (4.8)	
5	7 (1.9)	4 (4.4)	1 (0.8)	2 (1.9)	0 (0)	
Median delay to presentation (IQR) [minutes]	143 (74–294)	164 (86–307)	123 (69–265)	120 (68–248)	229 (95–487)	**0.02**
ICH etiology/classification (%)						
Deep (non-lobar), presumably hypertensive	156 (40.9)	35 (36.5)	59 (43.7)	44 (41.5)	18 (40.9)	0.28
Lobar	79 (20.7)	24 (25.0)	34 (25.2)	14 (13.2)	7 (15.9)	
OAC-associated	52 (13.7)	15 (15.6)	10 (7.4)	23 (21.7)	4 (9.1)	
Unknown	28 (7.3)	4 (4.2)	10 (7.4)	8 (7.6)	6 (13.6)	
Cavernoma	6 (1.6)	1 (1.0)	4 (3.0)	0 (0)	1 (2.3)	
Other vascular pathology^*^	22 (5.8)	7 (7.3)	6 (4.4)	6 (5.7)	3 (6.8)	
Metastasis/tumor	2 (0.5)	0 (0)	1 (0.7)	1 (0.9)	0 (0)	
Cerebral amyloid angiopathy	32 (8.4)	9 (9.4)	10 (7.4)	8 (7.6)	5 (11.4)	
Coagulation disorder	4 (1.1)	1 (1.0)	1 (0.7)	2 (1.9)	0 (0)	
Medical history (%)						
Arterial hypertension	307 (80.8)	82 (85.4)	112 (83.0)	80 (75.5)	33 (75.0)	0.2
Atrial fibrillation	73 (19.2)	21 (21.9)	24 (17.8)	24 (22.6)	4 (9.1)	0.22
Any type of stroke	73 (19.2)	23 (24.0)	23 (17.0)	17 (16.0)	10 (22.7)	0.41
Ischemic stroke	43 (11.3)	13 (13.5)	14 (10.4)	12 (11.3)	4 (9.1)	0.87
Hemorrhagic stroke	28 (7.4)	8 (8.3)	11 (8.2)	6 (5.7)	3 (6.8)	0.88
Smoking	57 (15.0)	15 (15.6)	13 (9.6)	17 (16.0)	12 (27.3)	**0.04**
Diabetes mellitus	57 (15.0)	14 (14.6)	18 (13.3)	14 (13.2)	11 (25.0)	0.29
Hypercholesterolemia	64 (16.8)	18 (18.8)	16 (11.9)	16 (15.1)	14 (31.8)	**0.03**
Alcohol	33 (8.7)	11 (11.5)	8 (5.9)	11 (10.4)	3 (6.8)	0.42

The groups are defined by the estimated ICH-onset time (morning: 5:00 AM−10:59 AM, midday: 11:00 AM−4:59 PM., evening: 5:00 PM−10:59 PM, night: 11:00 PM−4:59 AM).

The *p*-values < 0.05 that are considered statistically significant are depicted in a bold font.

^*^e.g., arteriovenous malformation, arteriopathy. ICH, intracerebral hemorrhage; IQR, Interquartile Range; mRS, Modified Rankin Scale; NIHSS, National Institutes of Health Stroke Scale; OAC, oral anticoagulation.

### Radiographic and functional outcomes

ICH and perifocal edema volume, mortality, stroke severity at presentation (NIHSS at admission and 24 h later) and global disability at discharge (mRS), showed no significant differences between the groups (Table 2).

**Table 2:** Primary and secondary outcomes according to the time of day of ICH onset.

Outcome measures	Morning (*n* = 96)	Midday (*n* = 135)	Evening (*n* = 106)	Night (*n* = 44)	*p*-value	IPW-adjusted *p*-value
Median ICH volume (in ml, IQR)	18.1 (4.3–44.8)	14.0 (5.4–44.8)	18.2 (4.8–50.5)	12.8 (3.3–28.1)	0.4	0.57
Median perilesional edema volume (in ml, IQR)	11.9 (4.8–38.8)	12.7 (6.2–30.0)	14.3 (5.7–34.5)	14.9 (3.8–29.7)	0.9	0.99
Mortality (%)	19 (19.8)	22 (16.3)	26 (24.5)	5 (11.4)	0.22	0.27
Presenting syndrome severity						
Median NIHSS at admission (IQR)	10 (4–20)	12 (4–20)	12 (5–26)	10 (5–17)	0.45	0.26
Severity classification (%)	–	–	–	–	0.71	0.58
Minor (NIHSS 0–3)	23 (24.0)	30 (22.6)	18 (17.0)	7 (15.9)	–	–
Moderate (NIHSS 4–10)	27 (28.1)	33 (24.8)	30 (28.3)	16 (36.4)	–	–
Severe (NIHSS 11–20)	24 (25.0)	38 (28.6)	26 (24.5)	13 (29.6)	–	–
Very severe (NIHSS >21)	22 (22.9)	32 (24.1)	32 (30.2)	8 (18.2)	–	–
15.6-7.4,-13.3499ptMedian NIHSS after 24 h (IQR)	11 (3–21)	7 (2–16)	10 (3–20)	8 (3–20)	0.67	0.46
Global disability at discharge						
Median mRS at discharge (IQR)	4 (3–5)	5 (3–5)	5 (3–5)	4 (3–5)	0.44	0.27
mRS (%)	–	–	–		0.64	0.48
0	3 (3.2)	4 (3.0)	3 (2.8)	1 (2.3)	–	–
1	8 (8.5)	13 (9.7)	8 (7.6)	4 (9.1)	–	–
2	9 (9.6)	12 (9.0)	9 (8.5)	5 (11.4)	–	–
3	11 (11.7)	12 (9.0)	10 (9.4)	2 (4.6)	–	–
4	23 (24.5)	20 (14.9)	15 (14.2)	11 (25.0)	–	–
5	21 (22.3)	51 (38.1)	35 (33.0)	16 (36.4)	–	–
6	19 (19.8)	22 (16.3)	26 (24.5)	5 (11.4)	–	–

ICH, intracerebral hemorrhage; IPW, inverse probability weighting; mRS, Modified Rankin Scale; NIHSS, National Institutes of Health Stroke Scale.

### Mortality is associated with age and syndrome severity, independent of time of onset

Given that mortality was numerically highest among individuals with evening onset, we evaluated whether time of day—specifically evening onset—served as an independent predictor of mortality using logistic regression analysis. The model identified increasing age (OR 1.04, 95% CI 1.01–1.07, *p* = 0.03), the pre-ICH mRS score (OR 1.37, 95% CI 1.04-1.81, *p* = 0.03) and greater syndrome severity, as measured by NIHSS at admission (OR 1.05, 95% CI 1.02–1.09, *p* = 0.002), as significant predictors of mortality. However, evening onset of ICH was not significantly associated with mortality (Table 3).

**Table 3:** Predictors of mortality at discharge (logistic regression model adjusted by inverse probability weighting, *R*^2^ Tjur 0.4).

Predictors	Odds ratios	95%-CI	*p*-value
Evening onset time	1.80	0.88–3.67	0.10
Delay to presentation	1.00	1.00–1.00	0.99
Age	1.04	1.01–1.07	**0.03**
Female sex	1.04	0.51–2.09	0.90
Pre-mRS	1.37	1.04–1.81	**0.03**
Arterial hypertension	2.38	0.86–7.20	0.11
Atrial fibrillation	1.51	0.68–3.33	0.31
Any type of stroke	0.68	0.13–2.93	0.62
Ischemic stroke	1.13	0.23–6.09	0.88
Hemorrhagic stroke	2.60	0.48–14.75	0.27
Smoking	0.63	0.15–2.10	0.49
Diabetes mellitus	2.01	0.82–4.82	0.12
Hypercholesterolemia	0.52	0.19–1.32	0.19
Alcohol	0.62	0.08–2.81	0.59
NIHSS at admission	1.05	1.02–1.09	**0.002**
Hematoma volume	1.01	0.99–1.02	0.34
Perifocal edema volume	1.02	1.00–1.03	0.08

mRS, Modified Rankin Scale; NIHSS, National Institutes of Health Stroke Scale.

The *p*-values < 0.05 that are considered statistically significant are depicted in a bold font.

Using an ordered multinomial regression, we further identified the following predictors of unfavorable outcomes (higher mRS scores at discharge): the pre-ICH mRS score (OR 1.33, 95% CI 1.19–1.48, *p* < 0.001), arterial hypertension (OR 1.49, 95% CI 1.06–2.10, *p* = 0.02), atrial fibrillation (OR 1.43, 95% CI 1.05–1.94, *p* = 0.02) and greater syndrome severity, as measured by NIHSS at admission (OR 1.06, 95% CI 1.04–1.07, *p* < 0.001). As previously observed, evening ICH onset was not predictive for the mRS at discharge (Supplementary Table S1).

## Discussion

This study found that ICH onset in patients treated at Frankfurt University Hospital follows a distinct daily pattern. In contrast to prior studies, it showed a somewhat later first incidence peak around noon (11–14) and a second peak around 4:00 PM. Only 11.5% of cases occurred at night, when hospital admission delays were also longest. Patients with ICH onset in the morning were older, while those with night-time onset showed higher rates of smoking and hypercholesterolemia. In our highly selective cohort, ICH and edema volumes were similar between onset groups and there were no differences in the initial syndrome severity and functional outcomes including intrahospital mortality. Consequently, time of day did not emerge as an independent predictor of mortality or poor functional outcome, these were instead linked to age, pre-ICH disability, initial NIHSS score, hypertension, and atrial fibrillation. So far, data on the impact of the time of day on ICH volumes and outcomes is conflicting. An analysis of the pooled INTERACT trials (including 2,904 patients) showed that daytime onset (8 am−4 pm) of ICH was associated with lesser clinical severity (as measured by presenting Glasgow Coma Scale), but no variation in initial hematoma volume or 90-day functional outcomes (15).

The Takashima Stroke Registry data from Japan showed an increased 30-day mortality in patients presenting in the morning (6:00 am−12 noon) (16), a study of 111 US American patients with spontaneous ICH found hematoma expansion occurred more frequently in patients presenting in daytime (8 am−8 pm) (17). Sreekrishnan and colleagues investigated differences in hematoma volume by image acquisition time in a large national database of automated ICH detection scans in over 19,000 patients. The analysis revealed that ICHs presenting at night were associated with larger ICH volumes compared to those cases imaged during the day or evening. Furthermore, the proportion of large ICHs (≥30 ml) was highest during nighttime hours (18). However, the study's reliance on imaging time as an indicator of symptom onset restricts the accuracy with which true daily onset patterns can be captured. The latter particularly applies to patients with night-time onset where imaging is likely to be further delayed. In the future, with an increasing widespread use of activity sensors (such as smart watches), onset-estimations may become more precise. Furthermore, the absence of patient characteristics and clinical outcome data restricts interpretation. By contrast, we precisely documented symptom onset times and presented our findings alongside data on clinical severity and short-term outcomes, including in-hospital mortality and overall disability. As a university hospital offering the highest level of care with 24-h neurosurgical availability, many cases were transfers for neurosurgical evaluation, which were typically conducted during office hours. Combined with the strict patient selection criteria (which excluded all patients with unknown onset times), this may have resulted in an overrepresentation of daytime cases and fewer cases in the nighttime group, ultimately impacting to answer our primary and secondary outcomes. Furthermore, we observed longer delays in night-time admissions. Given the rigorous determination of true symptom onset times in our study, and the observed prolongation of time to hospital admission during nighttime hours, the higher patient volumes observed in Sreekrishnan's study at night may be at least partly attributable to methodological biases, including inaccuracies in capturing true symptom onset and delays in downstream processes such as admission and imaging. This finding could inform emergency medical service and hospital triage protocols, as well as staffing, monitoring, and the allocation of resources during high- and low-risk periods (i.e., noon to afternoon and night, respectively).

This study has several limitations. First, the analysis was restricted to patients with a clearly documented stroke onset or last known well time, resulting in the exclusion of 70.7% of cases. A number which is higher compared to most acute stroke trials, usually ranging between 20 and 40% for unknown time windows (19–23) and potentially adding to a selection bias. Consequently, the baseline characteristics and outcomes of the excluded patients were not assessed. While this approach enables a more precise characterization of daily patterns in strokes with witnessed onset, it limits the generalizability of findings to strokes with uncertain or unwitnessed onset and might contributed to the low number of patients particularly in the night group.

Due to its retrospective design, this study lacks information on several chronobiologically relevant factors, such as individual chronotype, work schedules (shift work vs. standard daytime), whether stroke onset occurred on a workday or holiday, and the patient's activity at the time of onset. These variables are not typically captured in routine clinical practice and were therefore unavailable for analysis.

Additionally, given the hospital's proximity to an international airport, comparatively relevant proportion of patients presented after long-haul travel, which can disrupt circadian rhythms acutely. To reduce this confounding factor, such cases were excluded, which further added to the number of exclusions.

Our analysis focused on 24-h variations in stroke onset and did not consider other chronobiological influences, such as seasonal variation, which has previously been demonstrated in ICH patients (24, 25).

Our analysis focused solely on radiographic characteristics related to first imaging, without evaluating how timing may influence hemorrhage progression assessed by repeated imaging. Considering that the progression of the ischemic core in acute ischemic stroke may be influenced by the time of day (26, 27), this would be highly relevant. Previous studies have suggested that hematoma growth is more pronounced in patients admitted in the morning (15, 16), pointing to shared underlying mechanisms that contribute to secondary injury after acute ischemic and hemorrhagic stroke. Whether the time-of-day influences hematoma expansion remains unanswered, future studies targeting an impact on the course and outcomes of ICH are needed.

Outcome data were limited to the hospital stay (including intrahospital mortality and discharge mRS), which is insufficient to assess the long-term impact of ICH, given the typically prolonged recovery period. Furthermore, while our ordered multinominal regression model yielded a modest goodness of fit, it failed to well distinguish between the lower mRS scores 1–2 at discharge, most likely as these were underrepresented in our cohort. Prospective studies with extended follow-up and inclusion of chronobiologically relevant variables are therefore urgently needed to further assess the impact of the time of day on the clinical course and outcomes of ICH.

Despite the study's focus on a selective patient cohort, the findings provide evidence that ICH onset most frequently occurs during daytime hours, with no apparent impact on initial hematoma volume or the extent of perifocal edema. Early survival and short-term functional outcomes appear to be more strongly determined by individual patient factors than by the time of day. These results underscore the relevance of understanding daily patterns in ICH occurrence to enhance clinical response strategies and to better address modifiable risk factors in high-risk populations.

## Data Availability

The original contributions presented in the study are included in the article/supplementary material, further inquiries can be directed to the corresponding author.
