# Supplementary material for: Time-of-day variation affects onset but not hematoma size in intracerebral hemorrhage
Source: Front Neurol. 2026 Apr 13;17:1815359. doi: 10.3389/fneur.2026.1815359 (PMC13112484; doi:10.3389/fneur.2026.1815359)
Supplement: Supplementary file 1 [file Table_1.docx]

***Supplementary Material***

**1 Supplementary Tables**

| **Predictors** | **Odds Ratios** | **95%-CI** | **p value** |
| --- | --- | --- | --- |
| 0\|1 | 0.48 | 0.24 – 0.98 | **0.04** |
| 1\|2 | 1.29 | 0.67 – 2.50 | 0.45 |
| 2\|3 | 2.30 | 1.20 – 4.43 | **0.01** |
| 3\|4 | 3.83 | 1.98 – 7.40 | **<0.001** |
| 4\|5 | 7.97 | 4.07 – 15.61 | **<0.001** |
| 5\|6 | 30.70 | 14.98 – 62.92 | **<0.001** |
| Evening onset time | 1.10 | 0.85 – 1.43 | 0.46 |
| Delay to presentation | 1.00 | 1.00 – 1.00 | 0.10 |
| Age | 1.01 | 1.00 – 1.02 | 0.14 |
| Female sex | 1.15 | 0.90 – 1.47 | 0.28 |
| Pre-mRS | 1.33 | 1.19 – 1.48 | **<0.001** |
| Arterial hypertension | 1.49 | 1.06 – 2.10 | **0.02** |
| Atrial fibrillation | 1.43 | 1.05 – 1.94 | **0.02** |
| Any type of stroke | 0.59 | 0.34 – 1.02 | 0.06 |
| Ischemic stroke | 1.31 | 0.71 – 2.41 | 0.39 |
| Hemorrhagic stroke | 1.36 | 0.71 – 2.63 | 0.35 |
| Smoking | 0.86 | 0.61 – 1.20 | 0.37 |
| Diabetes mellitus | 0.94 | 0.67 – 1.31 | 0.71 |
| Hypercholesterolemia | 0.73 | 0.53 – 1.01 | 0.06 |
| Alcohol | 0.85 | 0.55 – 1.32 | 0.47 |
| NIHSS at admission | 1.06 | 1.04 – 1.07 | **<0.001** |
| Hematoma volume | 1.01 | 1.00 – 1.01 | 0.10 |
| Perifocal edema volume | 1.01 | 1.00 – 1.01 | 0.16 |

**Table S1:** Predictors of increasing mRS scores at discharge (ordered multinomial regression model adjusted by inverse probability weighting, R^2^ Nagelkerke 0.7).
